# Of Pompe and Circumstance: A Case Review of Late Onset Pompe Disease in a 52‐Year‐Old Male

**DOI:** 10.1155/crrh/8684758

**Published:** 2026-10-15

**Authors:** Jace Lehnhof, Jonathan Lin, Miten Vasa

**Affiliations:** ^1^ Scripps Mercy Internal Medicine Residency Program San Diego California USA; ^2^ Southern California Permanente Medical Group Fontana California USA kaiserpermanente.org

## Abstract

Late‐onset Pompe disease (LOPD) is a rare glycogen storage disorder caused by deficiency of the enzyme lysosomal acid alpha‐glucosidase (GAA). It can mimic inflammatory myopathies, leading to diagnostic delays. We present a case of LOPD in a 52‐year‐old man who came to us with a diagnosis of dermatomyositis for which he had been undergoing treatment for over a decade. Although he was on seemingly adequate immunosuppression, the patient continued to have progressive proximal muscular weakness during the first 4 years of our care. It was not until his sister was diagnosed with LOPD that we pursued biochemical and genetic testing that established the diagnosis by demonstrating reduced lysosomal acid alpha‐glucosidase activity and pathogenic variants in the GAA gene. The diagnosis of LOPD can be a clinical challenge given its rarity, as well as its similarities to other more common neuromuscular mimics. However, treatment is very different than myositis and is accomplished through enzyme replacement therapy, with the goal of preventing progression of the disease.

## 1. Introduction

Pompe disease was the first identified lysosomal storage disorder by Dutch pathologist Dr. Johannes Pompe in 1932, and late‐onset Pompe disease (LOPD) was first described by Dr. Andrew Engel in 1969. Also known as glycogen storage disease type II, it is a glycogen storage disease caused by a deficiency of the enzyme lysosomal acid alpha‐glucosidase (GAA), also called acid maltase. It is an autosomal recessive disorder caused by pathogenic variants in the gene for lysosomal acid alpha‐1,4 glucosidase. This enzymatic deficiency impairs lysosomal glycogen degradation, leading to progressive glycogen accumulation in lysosomes with eventual cytoplasmic spillover, resulting in autophagy dysregulation and subsequent tissue damage [1]. The degree of GAA activity determines the clinical presentation and time of disease onset. There is minimal to no GAA activity (< 1%) found in the infantile form and reduced activity (10%–30%) found in the late onset form [2]. Infantile Pompe disease classically presents with severe hypotonia, cardiomyopathy, macroglossia, hepatomegaly, failure to thrive, and ventilatory failure within the first few months of life [3]. On the other hand, LOPD does not typically cause cardiomyopathy but presents with progressive proximal muscle weakness, hypotonia, dysphagia, hyperlordosis, scoliosis, kyphosis, sleep disordered breathing, and diaphragmatic involvement, which can lead to exertional dyspnea and respiratory insufficiency [4, 5]. Given the severity of the infantile form, the US Department of Health and Human Services approved newborn screening for this disorder in 2015 [6]. As of this writing, 47 states and the District of Columbia were screened for Pompe disease in infants, making the patient’s age and state or country of birth important factors when considering this diagnosis.

The timeline to presentation for LOPD varies widely in surveys of affected individuals (0–62 years of age), with a mean age of symptom onset around 28 years old and a median age at diagnosis around 38 years old [7]. The onset of symptoms may be relevant to the prognosis as patients tend to do better with later onset of disease. The estimated prevalence of dermatomyositis is approximately 13–29 cases per 100,000, with incidence rates ranging from 1.1 to 3.0 per 100,000 person‐years [8, 9]. In contrast, LOPD is less common, with a global birth prevalence estimated at 2.4 cases per 100,000 live births [10]. The potential for immune‐mediated myopathies to coexist with and obscure LOPD has been reported, underscoring the importance of considering metabolic etiologies even when an autoimmune diagnosis has been established [11]. We present a case of LOPD diagnosed in an Egyptian man in the fifth decade of life, but who had been symptomatic since the third decade of life.

## 2. Case Presentation

A 52‐year‐old Egyptian man presented to our clinic with a history of dermatomyositis but was subsequently reclassified as having LOPD. At age 37, the patient developed muscle weakness in the proximal arms, legs, and neck, in conjunction with rashes on his hands and elbows. At age 41, he was diagnosed with dermatomyositis by an outside rheumatologist after electromyography (EMG), and left thigh muscle biopsy was reportedly consistent with myopathy. The actual reports of these tests were unavailable but were noted in the prior rheumatologist’s records.

He had been on various medication regimens, which included methotrexate, IVIG, azathioprine, and rituximab. On presentation, he was on prednisone 10 mg/day, methotrexate 15 mg/week, and azathioprine 100 mg/d. He reported generalized weakness and difficulty ambulating but denied dyspnea or rashes. Strength was reduced in the proximal upper (3+/5) and lower extremities (3‐/5), as well as in neck flexion/extension (3+/5). The patient also demonstrated bilateral foot drop, and his gait was weak and wide‐based. He required support from a chair to stand. Initial laboratory investigations showed grossly normal levels for CK, AST, ALT, aldolase, and LDH. A myositis‐specific antibody panel, ANA and SSA/SSB antibody levels, as well as ESR and CRP, were within normal limits.

We attributed the patient’s weakness to residual muscle damage, deconditioning, and possibly low‐level inflammation from myositis. We ordered a repeat EMG, muscle biopsy, MRI, and CT chest, abdomen, and pelvis. However, the patient’s follow‐up was sporadic, and he was initially nonadherent to the evaluations recommended, only receiving the CT scan, which demonstrated diffuse fatty infiltration and atrophy of the thoracic muscles (Figure 1). Throughout our care, his CK, AST, ALT, aldolase, and LDH levels were grossly normal.

**FIGURE 1:**
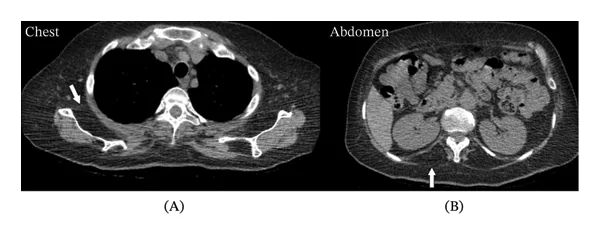
Noncontrast CT of chest and abdomen demonstrating diffuse fatty infiltration and atrophy of the thoracis muscles. (A) Symmetric severe fatty atrophy of the bilateral subscapularis muscles (arrow). (B) Symmetric severe fatty atrophy of the bilateral erector spinae muscles (arrow).

Four years after presentation, he reported that his sister was diagnosed with LOPD. Because the patient’s sister resided in a different state and received care through a different healthcare system, her medical records were not available for review. However, her diagnosis did prompt us to pursue biochemical and genetic testing. The patient demonstrated low GAA activity on a dried blood spot enzyme assay and tested positive for two pathologic variants of the GAA gene, both consistent with LOPD (Table 1). An MRI of the lower extremities performed at this time showed no evidence of muscle edema to indicate active inflammation but demonstrated symmetric muscle atrophy of the bilateral hips and thighs, consistent with Pompe disease (Figure 2). A geneticist confirmed the diagnosis, and a three‐generation family pedigree was evaluated, revealing no evidence of consanguinity. The patient was started on GAA enzyme replacement therapy (ERT) with Avalglucosidase alfa infusions with the goal of preventing the progression of weakness and related morbidity.

**TABLE 1:** Genetic and biochemical findings in the patient with late‐onset Pompe disease (LOPD).

Test	Result	Interpretation
GAA gene variants	Homozygous: c.‐32‐13T > G (intronic)	Pathogenic
	Heterozygous: c.1856G > A (p.Ser619Asn)	Pathogenic

GAA enzyme activity (DBS)	1.35 pmol/punch/hr	Decreased (Ref: > 3.88 pmol/punch/hr)

Urinary Hex4 level	4.5 mmol/mol creatinine	Elevated (Ref: ≤ 3 mmol/mol creatinine)

ACADS gene variant	Heterozygous: c.625G > A (p.Gly209Ser)	Nonpathogenic

RYR1 gene variant	Negative: c.1589G > A (p.Arg530His)	Not present

**FIGURE 2:**
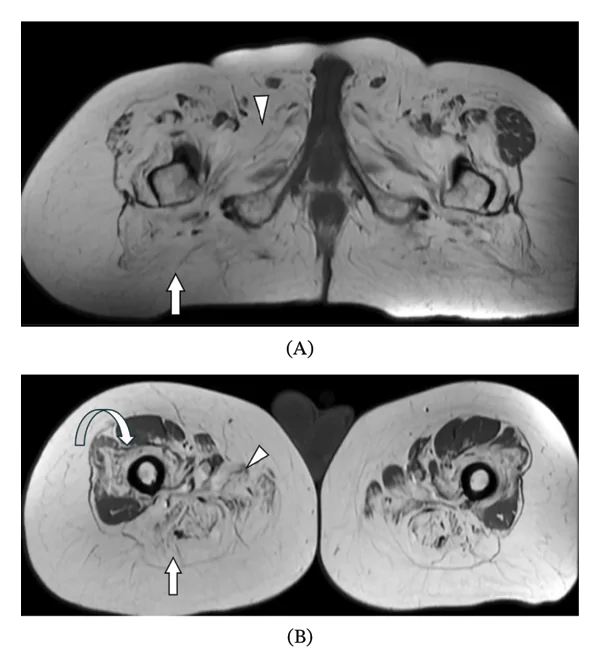
Lower extremity MRI axial T1‐weighted image of proximal thighs demonstrating fatty atrophy of legs. (A) Extensive symmetric fatty atrophy of the gluteus maximus muscles (arrow) and proximal adductor muscles (arrowhead). (B) Extensive symmetric fatty atrophy of the hamstring muscles (arrow), adductor muscles (arrowhead), and quadriceps muscles (curved arrow).

## 3. Discussion

LOPD is a rare autosomal recessive disorder caused by a mutation on the long arm of chromosome 17, the gene for lysosomal acid alpha‐1,4 glucosidase, resulting in reduced GAA activity due to enzyme deficiency. This leads to glycogen accumulation in the cytoplasm and lysosomes of tissues leading to muscle damage. In patients who have true objective proximal weakness and elevated CK, the prevalence of LOPD is estimated to be about 1.6% of the cases [12]. Consultation with a geneticist is invaluable as there are over 600 identified genetic variants, which must be differentiated from pseudodeficiency alleles and other related genetic conditions. Pseudodeficiency alleles are false‐positive alleles that cause low GAA enzyme activity without causing disease [13–15].

Clinically, LOPD has a subacute or chronic presentation, and the most commonly reported symptoms include weakness, hypotonia, shortness of breath, and fatigue [16]. Patients can have symmetric, proximal weakness that is typically more prominent in the lower extremities but can also affect upper extremities. Diaphragmatic involvement can lead to reduced forced vital capacity on pulmonary function tests, which may lead to respiratory infections, sleep apnea, and even respiratory failure. Depending on a patient’s pattern of muscular involvement, they could also experience difficulty chewing, dysphagia, decreased reflexes, hyperlordosis, and joint contractures [17–20].

The rarity of LOPD as well as its clinical similarities with other neuromuscular disorders presents a diagnostic challenge. The CK, aldolase, LDH, AST, and ALT levels could be variably elevated up to 10‐fold, with some cases even presenting as rhabdomyolysis. However, sometimes, these tests can be completely normal. Pompe disease used to be grouped under limb‐girdle muscular dystrophies until differences were identified, but other conditions in the differential diagnosis include glycogen storage disease type V and VI (McArdle’s and Her’s disease, respectively) and Duchenne–Becker muscular dystrophy [21]. The diagnosis of LOPD is often delayed with one case series of 17 patients reporting a mean of 8 years from symptom onset to diagnosis [22]. In our case, it took nearly 20 years from the patient’s first clinical symptoms until a definitive diagnosis was reached, partly due to an initial misdiagnosis of a mimicking condition 5 years after symptom onset, ambiguity in the medical record, and the discontinuity and irregularity of his presentation for medical care.

Our patient presented with proximal, painless muscle weakness, and the medical record also made mention of mechanic’s hands and possible Gottron’s papules on the hands and elbows, which could all be consistent with dermatomyositis. However, the cutaneous lesions were never present during our care. Inconsistent with dermatomyositis was the patient’s distal leg weakness and footdrop, which perhaps was related to his degenerative disc disease, as well as the progressive nature of his weakness despite normal CK and aldolase levels, negative myositis antibody panel (positive in 20%–40% of myositis cases), and seemingly adequate treatment for dermatomyositis.

Ambiguity in the medical records in this case included mention of a positive muscle biopsy and EMG studies consistent with myositis, for which formal reports were unable to be retrieved. In Pompe disease, EMG is nonspecific and can sometimes be normal, but it may also show irritable myopathy of proximal muscles, or myopathic discharges with abundant myotonic and complex repetitive discharges, most prominent in the axial and paraspinal muscles. Of note, spontaneous activity can be interpreted as “irritable myopathy,” which is typically seen in inflammatory or necrotizing myositis, which can lead to misdiagnosis [23]. Muscle biopsy is also nonspecific in Pompe disease and normal in up to 30%, but it may also resemble limb‐girdle muscular dystrophy or show glycogen storage within lysosomes and free glycogen in the cytoplasm on electron microscopy, as well as vacuolated fibers filled with glycogen with Period Acid‐Schiff stain positive vacuoles digestible by diastase and positive for acid phosphatase [24, 25]. Furthermore, MRI of the muscles is also nonspecific but can show atrophy of paravertebral and abdominal muscles, tongue, and hip extensor involvement, with sparing of lower leg muscles, deltoids, biceps, triceps, and a lack of muscle edema [26, 27]. Interestingly, hip extensor involvement and a lack of muscle edema did appear on our patient’s MRI of the lower extremities, and his CT scan did show diffuse fatty infiltration and atrophy of the thoracic muscles.

The prognosis of LOPD is better with later onset of symptoms, presumably due to less GAA enzyme deficiency [28]. In contrast, infantile Pompe disease is much more severe, and without treatment, there is only a reported 12%–26% survival at 12–18 months [29]. Some studies found that the odds of wheelchair use since diagnosis with LOPD increases by 13%/year, and the odds of respiratory support increases by 8%/year since diagnosis [30]. Five‐year survival rate without treatment is approximately 75% [31].

Should LOPD be suspected, diagnosis involves screening for GAA activity on dried blood spots, followed by confirmatory genetic testing. Treatment is with ERT with Avalglucosidase alfa infusions, which were first approved in 2006, with improved formulations subsequently approved in 2021 and 2023. ERT improves 5‐year survival to 95% [31]. In addition to mortality benefit, ERT can also delay the progression of muscle weakness and respiratory decline, demonstrating an increase in six‐minute walk test distances and improvement or stabilization of forced vital capacity. However, these benefits are most pronounced during the first 2‐3 years of treatment with efficacy declining over time [32]. To address the challenges posed by ERT, gene therapy is currently being studied in Phase 3 clinical trials, which would theoretically allow for a single treatment intervention for the disease [33]. Treatment should be multidisciplinary, involving genetics, physical therapy, occupational therapy, cardiology, and pulmonary specialists, as well as nutrition specialists.

This case highlights the challenges of inheriting a patient with a chronic diagnosis from a different health network, discontinuing long‐time treatments, and the difficult conversations of calling a long‐time diagnosis into question and requesting additional specialized testing and treatment. It underscores the importance of obtaining detailed medical records, regular patient follow‐up and continuity, and the need for confirmatory or repeat testing in the presence of diagnostic uncertainty. We were led to the correct diagnosis when new details to the patient’s family history became available. At the start of our care, the patient mentioned he had a sister reportedly with myositis, but she did not discover she had LOPD until 4 years into our care, thereby prompting investigation in our patient. This does demonstrate the importance of clarifying and investigating unusual family history such as two siblings with myositis, as well as obtaining a detailed and updated family history to elucidate rare genetic conditions such as LOPD.

## 4. Conclusion

LOPD is a rare genetic condition that can mimic other neuromuscular disorders including autoimmune inflammatory myopathies. The diagnosis of LOPD is a clinical challenge and often delayed by years from symptom onset. The diagnosis should be suspected in patients presenting with proximal weakness, similar to an inflammatory myopathy, but who, otherwise, do not have typical muscle enzyme elevations, typical myositis features found on EMG, MRI of the muscles, or muscle biopsy, or in whom muscle weakness continues to progress despite adequate treatment. Prompt diagnosis and early treatment with ERT can have a significant impact on patient outcomes and underscore the importance of awareness of LOPD in practitioners responsible for caring for patients with neuromuscular conditions.

## Funding

No funding was received for this manuscript.

## Consent

No written consent has been obtained from the patients as there are no patient identifiable data included in this case report.

## Conflicts of Interest

The authors declare no conflicts of interest.

## Data Availability

Data sharing is not applicable to this article as no datasets were generated or analyzed during the current study.
